# Exposure to screens and children’s language development in the EDEN mother–child cohort

**DOI:** 10.1038/s41598-021-90867-3

**Published:** 2021-06-08

**Authors:** Pauline Martinot, Jonathan Y. Bernard, Hugo Peyre, Maria De Agostini, Anne Forhan, Marie-Aline Charles, Sabine Plancoulaine, Barbara Heude

**Affiliations:** 1 Centre for Research in Epidemiology and StatisticS (CRESS), Université de Paris, Inserm, INRAE, 16 av Paul Vaillant-Couturier, 75004 Paris, France; 2 Singapore Institute for Clinical Sciences (SICS), Agency for Science, Technology and Research (A*STAR), Singapore, Singapore; 3 Laboratoire de Sciences Cognitives Et Psycholinguistique (ENS, EHESS, CNRS), Ecole Normale Supérieure, PSL Research University, Paris, France; 4 Neurodiderot, Inserm UMR 1141, Paris Diderot University, Paris, France; 5 Department of Child and Adolescent Psychiatry, Robert Debré Hospital, APHP, Paris, France

**Keywords:** Risk factors, Epidemiology, Paediatric research

## Abstract

Studies in children have reported associations of screen time and background TV on language skills as measured by their parents. However, few large, longitudinal studies have examined language skills assessed by trained psychologists, which is less prone to social desirability. We assessed screen time and exposure to TV during family meals at ages 2, 3 and 5–6 years in 1562 children from the French EDEN cohort. Language skills were evaluated by parents at 2 years (Communicative Development Inventory, CDI) and by trained psychologists at 3 (NEPSY and ELOLA batteries) and 5–6 years (verbal IQ). Cross-sectional and longitudinal associations were assessed by linear regression adjusted for important confounders. Overall, daily screen time was not associated with language scores, except in cross-sectional at age 2 years, where higher CDI scores were observed for intermediate screen time. Exposure to TV during family meals was consistently associated with lower language scores: TV always on (vs never) at age 2 years was associated with lower verbal IQ (− 3.2 [95% IC: − 6.0, − 0.3] points), independent of daily screen time and baseline language score. In conclusion, public health policies should better account for the context of screen watching, not only its amount.

## Introduction

Language is a thoroughly social phenomenon, and human interaction plays an important role in language acquisition^1^. Children’s language development is greatly influenced by their immediate environment (i.e., parents, siblings, and peers)^2^. Over the last decades, screens have become an additional part of the children’s environment, and preschool-aged children spend considerable time watching screens, especially TV^3,4^. Research has provided convincing evidence that it may lead to poorer cognitive and behavioral outcomes^5–7^, however, the observed effects largely depend on the age, the media content and the social and family context of viewing^5,7^. For example, high-quality media content and co-viewing with an adult may provide benefits for children’s learning and language skills^7,8^.

Although children are exposed to language via screen media, adult–child verbal interaction (questioning and commenting) is strongly associated with better children’s language development, which suggests that without such interaction or not using language in daily life to express themselves, children will not acquire a specific language^9,10^. Even when media programs are not intended for the child to watch, child–adult interaction and games are diminished when the TV is on^11^. Waldman et al. described the parents’ media behavior as an environmental trigger that creates hurdles in cognitive development with possible long-term developmental consequences^12^. Indeed, background TV was shown to reduce the amount and quality of interaction between parents and infants and the number of utterances produced by the parent–child dyad, with the direct effect of distracting a child and the indirect effect of taking a parent’s attention away from the child^9,11,13^.

However, there is limited evidence from large epidemiological studies on the role of the context of TV exposure on language development of pre-school children. In addition, daily screen time has been frequently, although not consistently, associated with language delays in infants and toddlers^2,6,9,14–18^. Disparate findings in the literature may be attributed to sampling differences due to small sample sizes, type of screen time measurement, and use of different language assessments. Moreover, most of these studies were cross-sectional and focused on children younger than age 3 years, which limits drawing conclusions on potential reverse causation. Furthermore, there is compelling evidence that screen time increases during early childhood^4,19^, and this change needs to be accounted for when analyzing longitudinal data. Lastly, few longitudinal studies have used specific psychological tests to assess language development and most studies rely on parental evaluation of the child’s language skills.

Analyzing data from the French mother–child EDEN cohort, we used both cross-sectional and longitudinal analyses to explore associations of daily screen time and exposure to TV during family meals with children’s language development at ages 2, 3 and 5–6 years while accounting for a wide range of established confounders and covariates.

## Methods

### Study design and population

**Figure 1:**
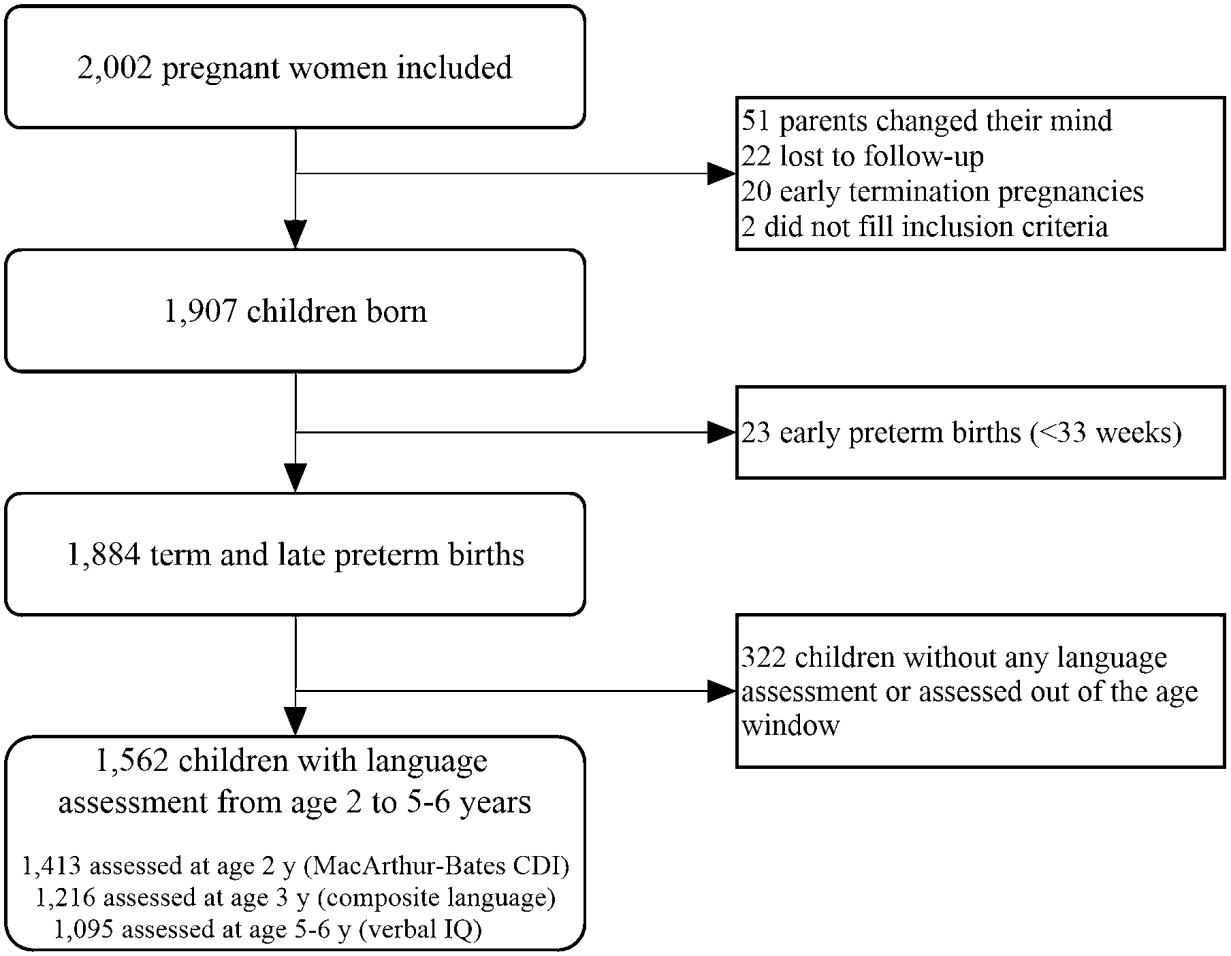
Flowchart of the EDEN participants analyzed in the present study. *CDI* communicative development inventory, *IQ* intelligence quotient.

Data were obtained from the EDEN mother–child study, a cohort aiming to identify prenatal and early postnatal nutritional, environmental, and social determinants of children's health and development. The study design and protocol have been published^20^. Briefly, 2002 pregnant women were enrolled between 2003 and 2006 in public maternity units of Poitiers and Nancy, France. A total of 1907 children were included and followed up. Exclusion criteria included a history of diabetes, twin pregnancies, intention to deliver outside the maternity unit or to move out of the study region within the next 3 years, and an inability to speak French. Children born at < 33 weeks’ gestation (n = 23) and children without any language assessment or assessed out of the assessment age window (n = 322) were excluded from the present work (Fig. 1). The study was approved by the Ethical Research Committee of Bicêtre Hospital and the French Data Protection Authority. Informed written consent was obtained from parents at the time of enrollment for themselves and for the newborn after delivery. All research was performed in accordance with the Declaration of Helsinki.

### Exposure to screens

Questionnaires were completed at ages 2, 3 and 5–6 years. Parents reported the time spent daily by their child watching TV or playing video or computer games on typical weekdays, Wednesdays (day off from school), and weekend days. Daily screen time was calculated as (4 × weekday + Wednesday + 2 × weekend day)/7. As the frequency distribution of daily screen time was multimodal (greater number of round values), we categorized it as 0, 1–30, 31–60, 61–120, and > 120 min at age 2 years. At ages 3 and 5–6 years, the categories 0 and 1–30 min were grouped because of insufficient number of children not watching screens at all. Exposure to TV during family meals was repeatedly assessed at the three ages with the following question: “How often is the television on in the dining room while the child is eating at home?” with four response items: never, sometimes, often, or always.

### Language assessment

When children were 2 years old, parents completed the French version of the MacArthur-Bates Communicative Development Inventory (CDI), an assessment of expressive vocabulary with high test–retest reliability and strong validity against the full version^21^. From a list of 100 words, parents indicated those their child could say spontaneously; and the sum was used as a score. At age 3 years, two trained psychologists (one per study center) assessed language development by using five neuropsychological subtests from the Evaluation du Langage Oral de L’enfant Aphasique^22^ and A Developmental NEuroPSYchological Assessment^23^ batteries: semantic fluency, word and nonword repetition, sentence comprehension task, sentence repetition, and picture naming. From these five subtest scores, a standardized composite language score was derived by principal component analysis with oblique rotation, as previously published^24^. In the present work, we re-scaled this variable (mean = 100, standard deviation = 15) so that subsequent effect sizes were more comparable with the verbal intelligence quotient (IQ) scale described below. At age 5–6 years (mean [± SD]: 67.9 [± 1.8] months), trained psychologists administered the French version of the Wechsler Preschool and Primary Scale of Intelligence-Third Edition^25^. The core subtests were assessed (information, vocabulary, word reasoning) to derive an age-adjusted verbal IQ.

### Covariates

Child sex, gestational age at birth, study center and birthweight were collected from obstetric records. Mothers completed questionnaires on their pre-pregnancy weight and tobacco and alcohol consumption during pregnancy. Maternal height was measured during pregnancy, allowing to calculate pre-pregnancy body mass index (BMI) as pre-pregnancy weight divided by height squared (in kg/m^2^) and classified according to the World Health Organization classification. Data on partial and exclusive breastfeeding duration were collected. Symptoms of postpartum depression were assessed with the Edinburgh Postnatal Depression Scale at 4, 8 and 12 months and were dichotomized with ≥ 12 as the threshold to define women at risk of depression.

Mothers and fathers completed questionnaires on their speech and language delay histories during childhood and on the frequency of shared cognitive activities with their child (e.g., reading stories, singing songs), both well-known predictors of language development^26^. At age 5–6 years, the quality of children's cognitive stimulation and emotional support in their home environment was assessed by the parents with items from the Home Observation for the Measurement of the Environment (HOME) scale^27^. Parents reported the date when their child entered preschool. In France, children can enter school from age 2 years, but most enter school within the year of their third birthday. Additional information on potential confounders collected included maternal age, household income, bilingual household, both parents’ education level, main caretaker, and number of older siblings aged < 14 years who were living at home. Parents reported children’s night sleep duration per 24 h, nap durations at ages 2 and 3 years, and sleep quality (night waking frequency per week). Frequent night waking was defined as ≥ 3 times per week.

### Statistical analyses

We describe the characteristics of participants with means (± SDs) and numbers (percentages) for continuous and categorical variables, respectively. We assessed the differences between excluded and included samples with Student *t* and chi-square tests.

We analyzed the associations of exposure to screens with language scores by multivariable linear regression. Analyzes were conducted both cross-sectionally (i.e., models with exposure to screens and outcome measured at concurrent time points) and longitudinally (i.e., models with exposure to screens measured at age 2 years and outcome measured at age 5–6 years). Longitudinal models were adjusted for language score at age 2 years (baseline) to estimate the effect of the exposure to screens at age 2 years on language at age 5–6 years while keeping baseline language constant. We adjusted regression models for the above-described confounders (except for sleep variables, see below). To gain precision in the prediction of the outcomes, we adjusted for the child’s exact age at language assessment (except for verbal IQ, which is already age-adjusted). We adjusted for schooling duration only in models at age 3 years, because, in our sample, almost no children had entered school at age 2 years, and almost all were in school at age 5–6 years. We evaluated the presence of multicollinearity between predictors of the models by calculating their variance inflation factor; as all were below 3, we considered multicollinearity not to be an issue in our models. We also examined the correlation between the two screen variables (Daily screen time and TV during family meals) and ran covariate-adjusted regression models with and without mutual adjustment for the two exposure variables to evaluate confounding by each other. We tested the interactions between daily screen time and TV during family meals but found none (data not shown).

To explore potential mediation by sleep^28^, we performed sensitivity analyses with further adjustment for sleep variables (night waking frequency, night sleep and nap durations) and observed whether it affected the magnitude of our estimates.

Missing data for exposures, outcomes and covariates were imputed by using multiple imputation techniques among the sample of participants with at least one measure of screen exposure and one measure of language (n = 1562) (Supplementary Table S4). We imputed 10 datasets by using the fully conditional specification method and combined the estimates following Rubin’s rules. We performed sensitivity analyses on the complete-case samples. Analyses were performed with SAS 9.3 (SAS Institute, Inc., Cary, NC, USA).

## Results

As compared with children excluded from our analyses, those included had a lower number of older siblings, lived in households of higher income and education level, and were born to mothers with lower pre-pregnancy BMI and less frequently smoked during pregnancy (Supplementary Table S1). The maternal, child and household characteristics of the 1,562 participants analyzed are shown in Table 1. Mean (± SD) daily screen time increased with age: 46 (± 47), 66 (± 50) and 84 (± 52) min at ages 2, 3 and 5–6 years, respectively (Table 2). The proportion of children never exposed to TV during family meals decreased from 40.5% to 37.8% and 34.1% from ages 2 to 3 and 5–6 years, respectively. Daily screen time and frequency of TV on during family meals were positively correlated with each other (Spearman correlations: 0.24, 0.31, 0.40 at ages 2, 3 and 5–6 years, respectively). Mean language scores were 60.7 (± 29.5) for CDI (age 2 years), 100.0 (± 15.0) (by construct) for composite language (3 years) and 106.7 (± 14.2) for verbal IQ (age 5–6 years) (Table 2).

**Table 1:** Maternal, child and household characteristics of the participants from the EDEN cohort.

	Observed sample (n = 1,562)^a,b^
**Maternal characteristics**	
Study center	
Poitiers	783 (50.1)
Nancy	779 (49.9)
Age at delivery, mean (SD), y	29.7 (4.8)
Pre-pregnancy body mass index	
< 18.5 kg/m^2^	128 (8.4)
18.5–24.9 kg/m^2^	1013 (66.2)
25.0–29.9 kg/m^2^	269 (17.6)
≥ 30.0 kg/m^2^	121 (7.9)
Tobacco consumption during pregnancy	373 (24.0)
Alcohol consumption during pregnancy	
None	868 (55.8)
< 2 glasses/week	573 (36.8)
≥ 2 glasses/week	117 (7.5)
Symptoms of postpartum depression	282 (21.7)
**Child characteristics**	
Sex	
Male	812 (52.0)
Female	750 (48.0)
Gestational age at birth, mean (SD), wk	39.4 (1.5)
Birthweight, mean (SD), kg	3.30 (0.47)
Breastfeeding duration, mean (SD), month	3.3 (3.7)
**Household characteristics**	
Older siblings	
0	718 (46.1)
1	566 (36.3)
≥ 2	275 (17.6)
Parental level of education, mean (SD), y	13.5 (2.3)
Bilingual household	132 (8.5)
Mother's language difficulties during childhood	93 (6.0)
Father's language difficulties during childhood	125 (8.7)
Monthly household income	
< 1,500 EUR	207 (13.3)
1,501–3,000 EUR	902 (57.7)
> 3,000 EUR	453 (29.0)

^a^Unless otherwise indicated, data are number (percentage) of participants.

^b^Data were missing for 31 participants (2%) for pre-pregnancy body mass index, 5 (0.3%) for tobacco consumption during pregnancy, 6 (0.4%) for alcohol consumption during pregnancy, 16 (1.0%) for symptoms of postpartum depression, 1 (0.1%) for birthweight, 1 (0.1%) for duration of breastfeeding, 3 (%) for older siblings, 22 (1.4%) for mother’s language difficulties, 131 (8.4%) for father’s language difficulties, 9 (0.6%) for household income.

**Table 2:** Children’s characteristics at ages 2, 3 and 5–6 years in the EDEN cohort^a^.

	Sample at age 2 years (n = 1,413)^b^	Sample at age 3 years (n = 1,216)	Sample at age 5–6 years (n = 1,095)
**Exposure to screens**			
Daily screen time, mean (SD), min	46 (47)	66 (50)	84 (52)
Daily screen time			
0 min	160 (12.2)	25 (2.2)	5 (0.5)
1–30 min	543 (41.3)	272 (23.8)	113 (11.3)
31–60 min	320 (24.3)	402 (35.2)	292 (29.1)
61–120 min	226 (17.2)	338 (29.6)	422 (42.0)
> 120 min	66 (5.0)	105 (9.2)	172 (17.1)
TV on during family meals			
Never	569 (40.5)	439 (37.8)	353 (34.1)
Sometimes	383 (27.3)	343 (29.5)	347 (33.5)
Often	284 (20.2)	238 (20.5)	225 (21.7)
Always	168 (12.0)	141 (12.1)	111 (10.7)
**Covariates**			
Main caretaker			
Nursery	314 (22.2)	229 (21.7)	351 (41.4)
Childminder	602 (42.6)	178 (16.9)	39 (4.6)
Family, neighbors	154 (10.9)	424 (40.2)	243 (28.7)
Mother	343 (24.3)	224 (21.2)	214 (25.3)
Cognitive stimulating activities shared with child			
< 1 time per week	14 (1.0)	13 (1.1)	122 (11.2)
1–2 times per week	467 (33.1)	470 (40.2)	201 (18.5)
3–5 times per week	207 (14.6)	215 (18.4)	424 (39.0)
Every day	725 (51.3)	471 (40.3)	185 (17.0)
HOME score	n.a	n.a	17.3 (2.3)
Schooling duration, mean (SD), month	n.a	2.9 (3.3)	n.a
Night sleep duration, mean (SD), hour	11.1 (0.8)	10.9 (0.7)	10.9 (0.5)
Nap duration, mean (SD), hour	2.1 (0.5)	1.9 (0.5)	n.a
Frequent night awakenings (≥ 3 times per week)	314 (22.3)	300 (25.8)	92 (9.0)
**Language development**			
Assessment tool	MacArthur-Bates CDI	Composite language	Verbal IQ
Exact age, mean (SD), month	23.8 (1.2)	37.3 (0.92)	67.2 (1.8)
Language score			
Mean (SD)	60.7 (29.5)	100.0 (15.0)	106.7 (14.2)
Median (interquartile range)	64 (35‒88)	102.6 (91.6‒110.5)	107 (98‒116)
Min‒max	1‒100	39.5‒133.7	44‒147

*CDI* Communicative Development Inventory, *HOME* Home Observation Measurement of the Environment, *IQ* intelligence quotient.

^a^Unless otherwise indicated, data are expressed number (percentage) of participants.

^b^Sample sizes indicated in column headers reflect largest sample size (i.e., the number of children with data for language development); data for exposure to TV and covariates were calculated without imputation for missing data and may be based on smaller sample sizes.

**Figure 2:**
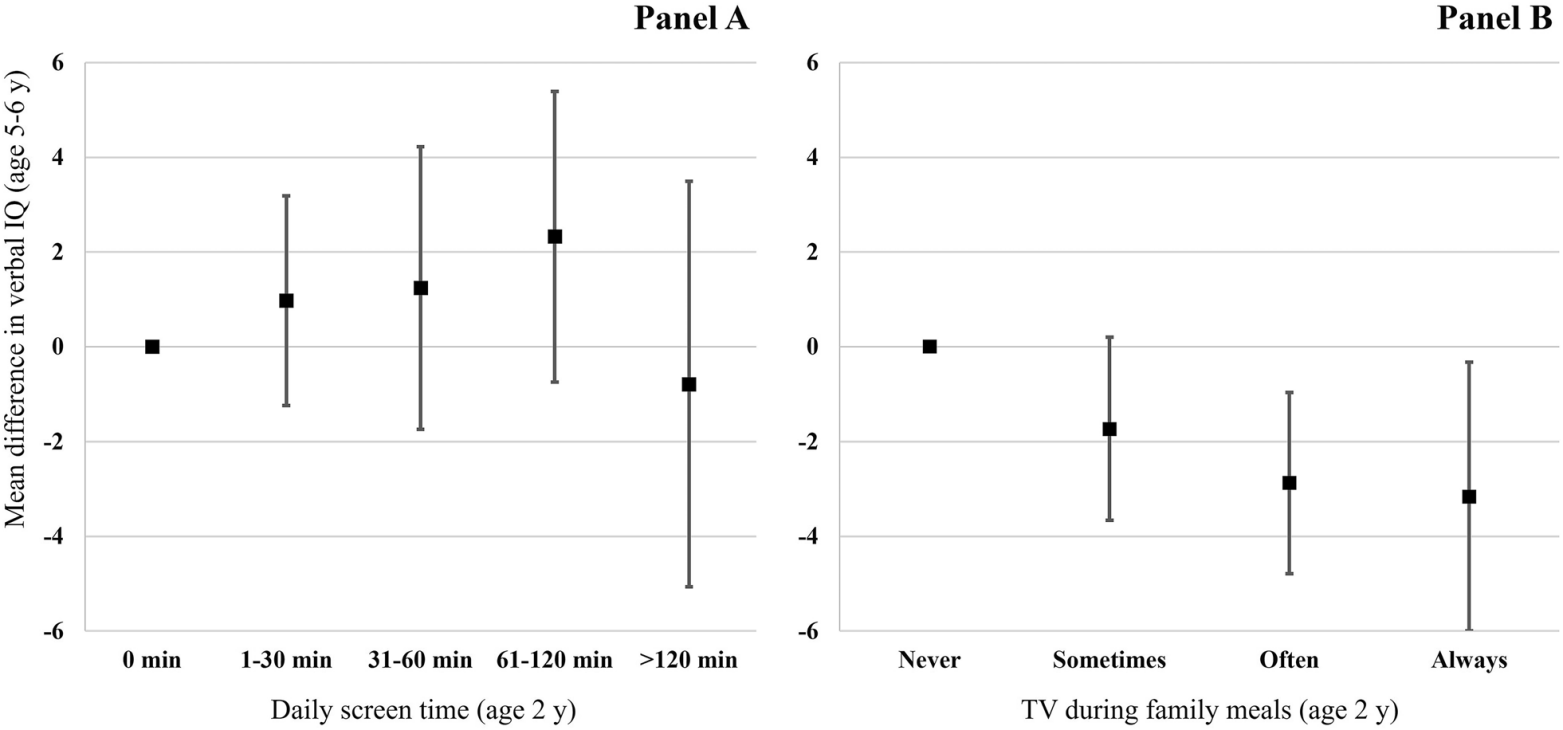
Mean difference in verbal IQ at age 5–6 years according to daily screen time (panel **A**) and exposure to TV during family meals (panel **B**) at age 2 years in the EDEN cohort. Error bars represent 95% confidence intervals around the mean difference estimates. P values for trend across categories are 0.60 and 0.002 for Panel A and Panel B, respectively. *IQ* intelligence quotient.

Table 3 shows the unadjusted and adjusted mean differences (and their 95% confidence interval) of the linear regression models assessing the associations between exposure to screens and language scores at concurrent time points (cross-sectional analyses). Of note, we observed an inverted U-shaped association between daily screen time and CDI score (age 2 years): the CDI score was 8.7 (95% CI: 3.4, 13.9) points higher on average for children watching screens for 31–60 min than never watching screens; CDI scores were intermediate for children watching screens for > 120 min daily. Although the shape of these associations also seemed nonlinear in cross-sectional analyses at ages 3 and 5–6 years (Table 3) and in the longitudinal analysis (ages 2 to 5–6 years; Fig. 2, Panel A), the associations were not statistically significant. Models with or without mutual adjustment for the two variables of exposure to screens carried out similar estimates (Supplementary Table S3).

**Table 3:** Unadjusted and adjusted cross-sectional associations between exposure to screens and child language development at each time point in the EDEN cohort (N = 1,562).^a^

	Cross-sectional models at age 2 years (MacArthur-Bates CDI as outcome)		Cross-sectional models at age 3 years (Composite language as outcome)		Cross-sectional models at age 5–6 years (Verbal IQ as outcome)	
	Unadjusted	Adjusted	Unadjusted	Adjusted	Unadjusted	Adjusted
**Daily screen time**						
0 min	0.0 (Reference)	0.0 (Reference)	–	–	–	–
1–30 min	6.8 (1.5, 12.2)	6.2 (1.2, 11.2)	0.0 (Reference)	0.0 (Reference)	0.0 (Reference)	0.0 (Reference)
31–60 min	7.6 (1.8, 13.3)	8.7 (3.4, 13.9)	0.6 (− 1.6, 2.9)	1.5 (− 0.5, 3.5)	0.2 (− 2.8, 3.2)	1.0 (− 1.8, 3.8)
61–120 min	6.8 (0.6, 13.1)	6.4 (0.7, 12.0)	− 0.6 (− 3.1, 2.0)	0.8 (− 1.5, 3.2)	− 0.0 (− 2.5, 2.5)	2.0 (− 0.6, 4.6)
> 120 min	1.7 (− 6.3, 9.8)	3.2 (− 4.8, 11.2)	− 2.6 (− 6.5, 1.3)	0.1 (− 3.6, 3.7)	− 1.6 (− 4.8, 1.6)	0.8 (− 2.4, 4.1)
**TV on during family meals**						
Never	0.0 (Reference)	0.0 (Reference)	0.0 (Reference)	0.0 (Reference)	0.0 (Reference)	0.0 (Reference)
Sometimes	− 2.4 (− 6.1, 1.4)	− 1.4 (− 4.9, 2.2)	− 4.8 (− 7.1, − 2.6)	− 2.8 (− 4.9, − 0.8)	− 5.0 (− 7.3, − 2.8)	− 3.3 (− 5.6, − 1.0)
Often	− 2.8 (− 7.0, 1.4)	− 0.7 (− 4.8, 3.4)	− 4.4 (− 6.8, − 2.1)	− 2.9 (− 5.2, − 0.6)	− 8.3 (− 11, − 6.0)	− 5.0 (− 7.4, − 2.7)
Always	− 13.7 (− 18.6, − 8.7)	− 6.7 (− 11.8, − 1.6)	− 9.8 (− 13, − 7.0)	− 4.6 (− 7.4, − 1.8)	− 6.9 (− 10, − 3.8)	− 2.5 (− 5.7, 0.7)

*CDI* Communicative Development Inventory, *IQ* intelligence quotient, *HOME* Home Observation Measurement of the Environment.

^a^Values are adjusted mean differences (vs the reference group) from linear regression models conducted on multiply imputed datasets (n = 1,562). Models are of cross-sectional design, i.e., outcomes are being predicted by exposure to screens as measured at concomitant age. Exposure to screens variables (Daily screen time and TV on during family meals) were mutually adjusted for each other. Adjusted models were further adjusted for the following covariates: study center, maternal age at delivery, pre-pregnancy body mass index, tobacco and alcohol consumption during pregnancy, symptoms of postpartum depression, child sex, gestational age at birth, birthweight, breastfeeding duration, number of older siblings, parental education level, bilingual household, maternal and paternal language difficulties during childhood, household income, main caretaker, cognitive stimulating activities, HOME score. Models at ages 2 and 3 years were further adjusted for the child’s exact age at language assessment (verbal IQ scoring accounts for age). Models at age 3 years were further adjusted for schooling duration.

Higher frequency of TV on during family meals was associated with poorer language scores in all cross-sectional (Table 3) and longitudinal (Fig. 2, Panel B) models. At age 2 years, the CDI score was lower for children always (vs never) exposed to TV during family meals (mean difference [95% CI]: − 5.9 [− 11.7, − 0.1] CDI points). In cross-sectional analyses at ages 3 and 5–6 years, language composite score and verbal IQ were higher for children never (vs sometimes or more frequently) exposed to TV during family meals (Table 3). In the longitudinal analysis, verbal IQ at age 5–6 years was lower for children always (vs never) exposed to TV during family meals at age 2 years (mean difference [95% CI]: − 3.2 [− 6.0, − 0.3] points). A dose–response relationship was observed for intermediate frequencies of TV during family meals (p for trend = 0.002) (Fig. 2, Panel B). Overall, multivariable adjustment did not substantially reduce the strengths of the associations seen in unadjusted models. Results with the complete-case analysis did not differ from those with multiple imputation (Supplementary Table S2). Furthermore, adjusting for children’s sleep quantity and quality did not significantly change the estimates (Supplementary Table S3).

## Discussion

In this cohort study of 1,562 children aged 2 to 5–6 years old, we found a cross-sectional inverted U-shaped association between children’s daily screen time at age 2 years and language development (with increased language scores for children watching screens for intermediate durations), but no associations at 3 or 5–6 years of age. In contrast, exposure to TV during family meals was negatively associated with language scores at every age, including when analyzing data longitudinally with adjustment for language skills at baseline.

Overall, we found no linear associations between daily screen time and language development, and this finding does not agree with the literature^7,16–18,29^. Rather, we found poorer language at age 2 years among children never watching screens, which may puzzle the readers. Previous studies have reported disparate results, which could be explained by residual confounding, and variations in study setting and language assessment methods. It is noteworthy that we found an association with the only language score that was evaluated by the parents, but not with those assessed by psychologists. However and in agreement with our results, another study found that infants exposed to no media actually had lower levels of language development than infants with some exposure^9^. Indeed, previous studies also suggested that parental characteristics and home environment (e.g., socioeconomic status and parental support) mattered more and were stronger predictors of children’s neurodevelopment than the amount of screen media use per se^10,30,31^. Another possible explanation for the inverted U-shape relationship could be that children watching moderate amount of TV watch programs of higher quality, and this may be beneficial for their language compared to non-watchers^32,33^.

Several studies have identified that human interaction, especially the frequency and quality of adults’ exchanges with their children, is crucial to children’s language development^7,34–37^. In agreement with other studies on TV exposure, the importance of child–adult interaction with regard to TV was reinforced when we explored the association between exposure to TV during family meals and language development^11,13,38,39^. Frequent exposure to TV during family meals was negatively associated with language scores at every age. Our main explanations for these findings, in line with previous work, are that TV during family meals may have both a direct effect of distracting a child and an indirect effect by taking a parent’s attention away from their child. In several studies, less verbal interaction with children when the TV was on were noted, as was less verbal production by children^7,10,13,15^. Also, auditive and visual stimulations may increase children’s and parents’ distractions in their family environment and increase the difficulties for a child to extract phonological and syntactical sounds from the background noise at home^40,41^. In agreement with these elements, we found that increased exposure to TV during family meals at age 2 years was strongly associated with poorer language at age 5–6 years.

Our study has several strengths, including its longitudinal design, large sample size and the availability of a wide range of confounding factors that few past studies were able to account for. Another important strength relies on the use of language tests assessed by trained psychologists; therefore, our findings are unlikely to be affected by social desirability bias arising from parental reporting only. Also, we performed longitudinal analyses of the associations between TV exposure in early childhood and later verbal IQ at age 5–6 years, a method ensuring that a potential cause precedes its potential effect, unlike cross-sectional analyses that have been the most frequent in the literature so far. Finally, we performed a sensitivity analysis with factors identified as potential mediators between TV exposure and language development: the results remained unchanged when including sleep characteristics, which agrees with recent results identifying a main action of sleep quality and quantity on executive functions rather than language development^42^.

Our study must be interpreted in light of some limitations. First, we could not examine screens other than TV and video games, such as smartphones and tablets, which have become increasingly widespread over the last decade. EDEN children turned 5–6 years between 2008 and 2012, when the market of handheld devices was only emerging and targeted adult users. Research in more recent cohorts is warranted. Second, we were not able to account for the content of children’s TV programs in our analysis. Lacking these data, we attempted to control for program content indirectly by including variables likely to be associated with the types of program a child watches (e.g., parental education, family income, parent–child interaction). Without direct adjustment for TV content programs, residual confounding may remain, however. Third, we measured children’s exposure to screens with parent-reported questionnaires; this method is relatively inaccurate and suffers from social desirability. Future studies need more objective and comprehensive methods for measuring screen use to tackle this limitation^43^. Finally, the three language assessments we conducted were not directly comparable, which limits our ability to implement models with repeated measures.

Despite 2016 American Academy of Pediatrics’ recommendations^5^, as well as European scientific academic reports suggesting thresholds on age limits or TV time for children, we lack evidence-based consensus. Families need to be better informed about what activities really promote their children’s healthy neurodevelopment. This work consolidates previous results and adds new elements to support recommendations, especially with regard to the context of TV viewing.

In this analysis, we found no relationship between daily screen time and language development, except cross-sectionally at age 2 years with a U-shaped relationship where children exposed to TV for intermediate times had greater scores. We found, however, consistent negative dose–response associations between frequency of exposure to TV during family meals and language development. Our findings encourage scientists and decision-makers to better consider contextual traits of screen viewing.

## Supplementary Information

Supplementary Information.

## Data Availability

The datasets generated or analyzed during the current study are not publicly available due to ethical restrictions related to protecting patient confidentiality and legal restrictions imposed by the French National Commission on Data Processing and Liberties (CNIL). Investigators who wish to access the data reported in this article must address a reasonable request to the EDEN steering committee at etude.eden@inserm.fr.

## Abbreviations

CDI: Communicative Development Inventory
EDEN: Etude des Déterminants pré et postnatals précoces du développement de la santé de l’Enfant [Study of the Early Pre and Postnatal Determinants of Childhood Health Development]
IQ: Intelligence quotient
