# Supplementary material for: Exposure to screens and children’s language development in the EDEN mother–child cohort
Source: Sci Rep. 2021 Jun 8;11:11863. doi: 10.1038/s41598-021-90867-3 (PMC8187440; doi:10.1038/s41598-021-90867-3)
Supplement: Supplementary file 1 — Supplementary Information. [file 41598_2021_90867_MOESM1_ESM.pdf]

Supplementary Information

Exposure to Screens and Children's Language Development in the EDEN Mother–Child Cohort

Pauline Martinot[1], MD, Jonathan Y. Bernard[1,2]*, PhD, Hugo Peyre[3,4,5], MD, PhD, Maria De Agostini[1], PhD, Anne Forhan[1], MSc, Marie-Aline Charles[1], MD, Sabine Plancoulaine[1], MD, PhD, Barbara Heude[1], PhD

Affiliations:

[1] Université de Paris, Centre for Research in Epidemiology and StatisticS (CRESS), Inserm, INRAE, F-75004 Paris, France;

[2] Singapore Institute for Clinical Sciences (SICS), Agency for Science, Technology and Research (A*STAR), Singapore, Singapore;

[3] Laboratoire de Sciences Cognitives et Psycholinguistique (ENS, EHESS, CNRS), Ecole Normale Supérieure, PSL Research University, Paris, France ;

[4] Neurodiderot, Inserm UMR 1141, Paris Diderot University, Paris, France;

[5]Department of Child and Adolescent Psychiatry, Robert Debré Hospital, APHP, Paris, France.

PM and JYB are joint first authors; SP and BH are joint senior authors. *JYB is corresponding author.

List of supplementary tables:

Supplementary Table S1. Comparison of the included and excluded samples of participants from the EDEN cohort.

Supplementary Table S2. Adjusted associations between exposure to screens and child language development in the EDEN cohort - complete-case analysis with or without mutual adjustment for exposure to screens variables.

Supplementary Table S3. Adjusted cross-sectional and longitudinal associations between exposure to screens and child language development in the EDEN cohort - sensitivity analysis with further adjustment for child sleep quality and quantity (N=1,562).

Supplementary Table S4. Characteristics of the variables used for multiple imputation.

Supplementary Information

Supplementary Table S1. Comparison of the included and excluded samples of participants from the EDEN cohort.

	Excluded sample (n=322)	Included sample (n=1,562)[a,b]	P-value
**Maternal characteristics**			
Study center			0.14
Poitiers	147 (45.7)	783 (50.1)	
Nancy	175 (54.3)	779 (49.9)	
Age at delivery, mean (SD), y	28.2 (±5.2	29.7 (4.8)	<0.0001
Pre-pregnancy body mass index			0.04
<18.5 kg/m^2	33 (10.5)	128 (8.4)	
18.5-24.9 kg/m^2	189 (60.2)	1013 (66.2)	
25.0-29.9 kg/m^2	54 (17.2)	269 (17.6)	
≥30.0 kg/m^2	38 (12.1)	121 (7.9)	
Tobacco consumption during pregnancy	134 (41.6)	373 (24.0)	<0.0001
Alcohol consumption during pregnancy			0.07
None	199 (62.6)	868 (55.8)	
<2 glasses/week	92 (28.9)	573 (36.8)	
≥2 glasses/week	27 (8.5)	117 (7.5)	
Symptoms of postpartum depression	6.2 (5.0)	4.9 (4.0)	0.0007
**Child characteristics**			
Sex			0.32
Male	175 (55.0)	812 (52.0)	
Female	143 (45.0)	750 (48.0)	
Gestational age at birth, mean (SD), wk	39.2 (1.5)	39.4 (1.5)	0.17
Birthweight, mean (SD), kg	3.29 (0.5)	3.30 (0.47)	0.61
Breastfeeding duration, mean (SD), month	2.6 (3.0)	3.3 (3.7)	0.0002
**Household characteristics**			
Older siblings			0.002
0	116 (36.3)	718 (46.1)	
1	126 (39.4)	566 (36.3)	
≥2	78 (24.4)	275 (17.6)	
Parental level of education, mean (SD), y	12.3 (2.3)	13.5 (2.3)	<0.0001
Bilingual household	2 (0.6)	132 (8.5)	<0.0001
Mother's language difficulties during childhood	32 (10.1)	93 (6.0)	0.009
Father's language difficulties during childhood	20 (7.8)	125 (8.7)	0.64
Monthly household income			<0.0001
<1,500 EUR	101 (31.4)	207 (13.3)	
1,501-3,000 EUR	163 (50.6)	902 (57.7)	
>3,000 EUR	58 (18.0)	453 (29.0)	

[a] Unless otherwise indicated, data are number (percentage) of participants.

[b] Data were missing for 31 participants (2%) for pre-pregnancy body mass index, 5 (0.3%) for tobacco consumption during pregnancy, 6 (0.4%) for alcohol consumption during pregnancy, 16 (1.0%) for symptoms of postpartum depression, 1 (0.1%) for birthweight, 1 (0.1%) for duration of breastfeeding, 3 (%) for older siblings, 22 (1.4%) for mother's language difficulties, 131 (8.4%) for father's language difficulties, 9 (0.6%) for household income.

Supplementary Table S2. Adjusted associations between exposure to screens and child language development in the EDEN cohort - complete-case analysis with or without mutual adjustment for exposure to screens variables.[a]

	Cross-sectional models at age 2 years (MacArthur-Bates CDI as outcome)	Cross-sectional models at age 3 years (Composite language as outcome)	Cross-sectional models at age 5-6 years (Verbal IQ as outcome)	Longitudinal models from age 2 (exposure to screens) to 5-6 years (verbal IQ)
**N**	1,154	998	856	759
**Single-exposure models**				
Daily screen time				
0 min	0.0 (Reference)	-	-	0.0 (Reference)
1-30 min	6.7 (1.7, 11.8)	0.0 (Reference)	0.0 (Reference)	1.2 (-1.6, 4.0)
31-60 min	8.0 (2.5, 13.5)	1.7 (-0.4, 3.9)	0.9 (-2.1, 3.9)	0.9 (-2.1, 3.9)
61-120 min	5.6 (-0.4, 11.5)	-0.5 (-2.8, 1.8)	0.8 (-2.2, 3.7)	1.4 (-1.9, 4.7)
>120 min	2.0 (-6.5, 10.5)	-0.9 (-4.3, 2.5)	-0.4 (-4.0, 3.1)	-3.1 (-8.1, 1.8)
TV on during family meals				
Never	0.0 (Reference)	0.0 (Reference)	0.0 (Reference)	0.0 (Reference)
Sometimes	-0.4 (-4.3, 3.5)	-3.3 (-5.5, -1.2)	-3.3 (-5.4, -1.2)	-2.2 (-4.4, -0.0)
Often	0.4 (-4.0, 4.9)	-2.9 (-5.3, -0.4)	-5.0 (-7.5, -2.5)	-3.9 (-6.3, -1.5)
Always	-6.4 (-11.9, -0.9)	-5.1 (-8.1, -2.0)	-2.7 (-6.0, 0.6)	-3.6 (-6.6, -0.6)
**Mutually-adjusted model**				
Daily screen time				
0 min	0.0 (Reference)	-	-	0.0 (Reference)
1-30 min	6.6 (1.5, 11.6)	0.0 (Reference)	0.0 (Reference)	1.5 (-1.2, 4.3)
31-60 min	7.9 (2.3, 13.4)	2.1 (-0.1, 4.2)	1.9 (-1.1, 4.9)	1.7 (-1.3, 4.7)
61-120 min	5.9 (-0.1, 11.9)	0.1 (-2.2, 2.5)	2.3 (-0.7, 5.3)	2.3 (-1.1, 5.6)
>120 min	2.3 (-6.3, 10.9)	0.2 (-3.3, 3.7)	1.3 (-2.3, 4.9)	-1.9 (-6.9, 3.1)
TV on during family meals				
Never	0.0 (Reference)	0.0 (Reference)	0.0 (Reference)	0.0 (Reference)
Sometimes	-1.3 (-5.4, 2.7)	-3.7 (-5.8, -1.5)	-3.9 (-6.1, -1.7)	-2.8 (-5.1, -0.5)
Often	-0.0 (-4.6, 4.6)	-3.2 (-5.8, -0.7)	-5.6 (-8.2, -3.0)	-4.1 (-6.7, -1.5)
Always	-5.9 (-11.7, -0.1)	-5.0 (-8.1, -1.9)	-3.1 (-6.5, 0.4)	-3.6 (-6.8, -0.5)

Abbreviations: CDI, Communicative Development Inventory; HOME, Home Observation Measurement of the Environment; IQ, intelligence quotient.

[a] Values are adjusted mean differences (vs the reference group), resulting from linear regression models conducted on complete-case samples. In cross-sectional models, the outcome is predicted by exposure to screens as measured at concomitant age. In the longitudinal models, the outcome at age 5-6 years is predicted by exposure to screens measured at age 2 years, with adjustment for language at 2 years. Single-exposure models are independent models where a single exposure to screens variable is included (either Daily screen time, either TV on during family meals); mutually-adjusted models include both variables simultaneously. Models were adjusted for the following covariates: study center, maternal age at delivery, pre-pregnancy body mass index, tobacco and alcohol consumption during pregnancy, symptoms of postpartum depression, child sex, gestational age at birth, birthweight, breastfeeding duration, number of older siblings, parental education level, bilingual household, maternal and paternal language difficulties during childhood, household income, main caretaker, cognitive stimulating activities, HOME score. Models at ages 2 and 3 years were further adjusted for the child's exact age at language assessment (verbal IQ scoring procedure accounts for age). Models at age 3 years were further adjusted for schooling duration.

Supplementary Table S3. Adjusted cross-sectional and longitudinal associations between exposure to screens and child language development in the EDEN cohort - sensitivity analysis with further adjustment for child sleep quality and quantity (N=1,562).[a]

	**Cross-sectional models at age 2 years (MacArthur-Bates CDI as outcome)**	**Cross-sectional models at age 3 years (Composite language as outcome)**	**Cross-sectional models at age 5-6 years (Verbal IQ as outcome)**	**Longitudinal model from age 2 (exposure to screens) to 5-6 years (verbal IQ)**
Daily screen time				
0 min	0.0 (Reference)	-	-	0.0 (Reference)
1-30 min	6.1 (1.1, 11.1)	0.0 (Reference)	0.0 (Reference)	1.0 (-1.3, 3.2)
31-60 min	8.6 (3.3, 13.8)	1.5 (-0.4, 3.5)	1.0 (-1.8, 3.8)	1.3 (-1.8, 4.3)
61-120 min	6.1 (0.3, 11.8)	0.8 (-1.6, 3.1)	2.1 (-0.5, 4.6)	2.3 (-0.8, 5.5)
>120 min	2.3 (-5.7, 10.4)	0.3 (-3.3, 3.9)	0.9 (-2.3, 4.1)	-0.8 (-5.1, 3.4)
TV on during family meals				
Never	0.0 (Reference)	0.0 (Reference)	0.0 (Reference)	0.0 (Reference)
Sometimes	-1.4 (-5.0, 2.2)	-2.8 (-4.9, -0.8)	-3.3 (-5.6, -1.0)	-1.7 (-3.7, 0.2)
Often	-0.9 (-5.0, 3.2)	-2.9 (-5.2, -0.5)	-5.0 (-7.4, -2.7)	-2.9 (-4.8, -0.9)
Always	-6.9 (-12.0, -1.7)	-4.4 (-7.2, -1.7)	-2.4 (-5.7, 0.8)	-3.1 (-5.9, -0.4)

Abbreviations: CDI, Communicative Development Inventory; HOME, Home Observation Measurement of the Environment; IQ, intelligence quotient.

[a] Values are adjusted mean differences (vs the reference group), resulting from linear regression models conducted on multiply imputed datasets (n=1,562). In cross-sectional models, the outcome is predicted by exposure to screens as measured at concomitant age. In the longitudinal model, the outcome at age 5-6 years is predicted by exposure to screens measured at age 2 years, with adjustment for language at 2 years. Exposure to screens variables (Daily screen time and TV on during family meals) were mutually adjusted for each other. Exposure to screens variables (Daily screen time and TV on during family meals) were mutually adjusted for each other. Models were further adjusted for the following covariates: study center, maternal age at delivery, pre-pregnancy body mass index, tobacco and alcohol consumption during pregnancy, symptoms of postpartum depression, child sex, gestational age at birth, birthweight, breastfeeding duration, number of older siblings, parental education level, bilingual household, maternal and paternal language difficulties during childhood, household income, main caretaker, cognitive stimulating activities, HOME score. Models at ages 2 and 3 years were further adjusted for the child's exact age at language assessment (verbal IQ scoring procedure accounts for age). Models at age 3 years were further adjusted for schooling duration.

Supplementary Information

Supplementary Table S4. Characteristics of the variables used for multiple imputation.

Variable	Type of variable	Model used to predict missing data	Missing (n)	Missing (%)
Bilingual household	Binary	No missing data	0	0.0
Child sex	Binary	No missing data	0	0.0
Gestational age, week	Continuous	No missing data	0	0.0
Maternal age at delivery	Continuous	No missing data	0	0.0
Recruitment center	Binary	No missing data	0	0.0
Breastfeeding duration, month	Continuous	Linear regression	1	0.1
Child birthweight, kg	Continuous	Linear regression	1	0.1
Number of older siblings	Continuous	Linear regression	3	0.2
Tobacco consumption during pregnancy	Binary	Logistic regression	5	0.3
Alcohol consumption during pregnancy	Continuous	Linear regression	6	0.4
Household income, euros/month	Continuous	Linear regression	9	0.6
Maternal education level, y	Continuous	Linear regression	9	0.6
Symptoms of postpartum depression	Continuous	Linear regression	16	1.0
Main caretaker (age 2 y)	Categorical	Multinomial regression	19	1.2
Shared cognitive activities with child (age 2 y)	Continuous	Linear regression	19	1.2
Mother's language difficulties during childhood	Binary	Logistic regression	22	1.4
Mother's pre-pregnancy body mass index	Categorical	Multinomial regression	31	2.0
Father's language difficulties during childhood	Binary	Logistic regression	131	8.4
Paternal education level, y	Continuous	Linear regression	134	8.6
Child’s exact age when assessing questionnaire (age 2 y)	Continuous	Linear regression	141	9.0
MacArthur-Bates CDI score (age 2 y)	Continuous	Linear regression	149	9.5
Frequent night awakenings (age 2 y)	Binary	Logistic regression	150	9.6
TV during family meals (age 2 y)	Categorical	Multinomial regression	157	10.0
Nap duration (age 2), hour	Continuous	Linear regression	186	12.0
Daily screen time (age 2 y)	Categorical	Multinomial regression	247	16.0
Frequent night awakenings (age 3 y)	Binary	Logistic regression	271	17.0
Night sleep duration (age 2 y), hour	Continuous	Linear regression	273	17.0
Schooling duration (age 3 y), month	Continuous	Linear regression	265	17.0
Shared cognitive activities with child (age 3 y)	Continuous	Linear regression	268	17.2
TV during family meals (age 3 y)	Categorical	Multinomial regression	276	18.0
Daily screen time (age 3 y)	Categorical	Multinomial regression	302	19.0
Child’s exact age when assessing questionnaire (age 3 y)	Continuous	Linear regression	334	21.0
HOME score (age 5-6 y)	Continuous	Linear regression	340	22.0
Night sleep duration (age 3 y), hour	Continuous	Linear regression	348	22.0
Composite language score (age 3 y)	Continuous	Linear regression	346	22.2
TV during family meals (age 5-6 y)	Categorical	Multinomial regression	390	25.0
Frequent night awakenings, (age 5-6 y)	Binary	Logistic regression	402	26.0
Nap duration (age 3 y), hour	Continuous	Linear regression	402	26.0
Night sleep duration (age 5-6 y), hour	Continuous	Linear regression	413	26.0
Daily screen time (age 5-6 y), hour	Categorical	Multinomial regression	426	27.0
Father’ cognitive stimulating activities shared with child (age 5-6 y)	Continuous	Linear regression	432	28.0
Verbal IQ (age 5-6 y)	Continuous	Linear regression	467	30.0
Main caretaker (age 3 y)	Categorical	Multinomial regression	496	32.0
Main caretaker (age 5 y)	Categorical	Multinomial regression	604	39.0

Abbreviations: CDI, Communicative Development Inventory; HOME, Home Observation Measurement of the Environment; IQ, intelligence quotient.
